# *Chinese Medicine* reviewer acknowledgement 2015

**DOI:** 10.1186/s13020-016-0082-0

**Published:** 2016-03-31

**Authors:** Siu-wai Leung

**Affiliations:** International Society for Chinese Medicine, University of Macau, Macao, China; State Key Laboratory of Quality Research in Chinese Medicine, Institute of Chinese Medical Sciences, University of Macau, Macao, China

## Contributing reviewers

The Editors of *Chinese Medicine* would like to thank all our reviewers who have contributed to the journal in volume 10 (2015).

**Rana Abu-Dahab**

Jordan

**Noorjahan Banu Alitheen**

Malaysia

**Nicolino Ambrosino**

Italy

**Rachel Claire Anderson**

New Zealand

**Minetaro Arita**

Japan

**Vasumathi Asha**

India

**Niranjan Awasthi**

USA

**Gang Bai**

China

**Thereza Bargut**

Brazil

**Huveyda Basaga**

Turkey

**Zhaoxiang Bian**

Hong Kong

**Peng Cao**

China

**Alan Carne**

New Zealand

**Sergio Catanozi**

Brazil

**Thomas Chan**

Hong Kong

**Hsueh-Wei Chang**

Taiwan

**Raymond Chuen-Chung Chang**

Hong Kong

**Chun-Tao Che**

USA

**Ke-Ming Chen**

China

**Sibao Chen**

China

**Haifeng Chen**

China

**Yu Chen**

China

**Yuezhou Chen**

USA

**Jia-Xu Chen**

China

**Chieh-Fu Chen**

Taiwan

**Hubiao Chen**

Hong Kong

**Weiwei Chen**

China

**Juei-Tang Cheng**

Taiwan

**Fan Cheung**

Hong Kong

**Jae Youl Cho**

South Korea

**Vincent Chung**

Hong Kong

**Hau Yin Chung**

Hong Kong

**Kieran Cooley**

Canada

**Zeynep Coskun**

Turkey

**Zheng-Guo Cui**

Japan

**Yuchuan Ding**

USA

**Lijun Du**

China

**Jin Ao Duan**

China

**Peter Eckl**

Austria

**Thomas Efferth**

Germany

**Thomas Eidenberger**

Austria

**Daping Fan**

USA

**Evandro Fang**

USA

**Yibin Feng**

Hong Kong

**Arthur Sa Ferreira**

Brazil

**Fuyou Fu**

USA

**Po-Wu Gean**

Taiwan

**Peter Gerard Gibson**

Australia

**Ingrid Glurich**

USA

**William Goggins**

Hong Kong

**Adel Gomaa**

Egypt

**Fulan Guan**

USA

**Gunjan Guha**

India

**Jianming Guo**

China

**Young Gwak**

South Korea

**Simon Han**

Hong Kong

**Jianping Han**

China

**Swei Sunny Hann**

China

**Shanshan He**

USA

**Chengwei He**

Macao

**Yong Heo**

South Korea

**Po-Jen Hsiao**

Taiwan

**Pei-Wen Hsiao**

Taiwan

**Ching-Liang Hsieh**

Taiwan

**Hao Hu**

Macao

**Linfang Huang**

China

**Shuang Huang**

USA

**Thanasekaran Jayakumar**

Taiwan

**Kee Ching Jeng**

Taiwan

**Guang Ji**

Ghana

**Zhi-Hong Jiang**

China

**Lan Jiang**

USA

**Henry J. Kaplan**

USA

**Amber Keizerweerd**

USA

**Thomas Kelly**

USA

**Taro Kishida**

Japan

**Kam Ming Ko**

Hong Kong

**Yuske Komiyama**

Japan

**Ah-Ng Tony Kong**

USA

**Yun Hyung Koog**

South Korea

**Nils Kraehmer**

Germany

**Haixue Kuang**

China

**Enoch Kumar**

Malaysia

**Hoi-Hin Kwok**

Hong Kong

**Gilles Labbe**

France

**Kristina Lamas**

Sweden

**Tai Wai David Lau**

Hong Kong

**Simon Ming Yuen Lee**

Macao

**Bombi Lee**

South Korea

**I. Michael Leitman**

USA

**Siu-wai Leung**

Macao

**Jie Li**

USA

**Yaolan Li**

China

**Xican Li**

China

**Shao Li**

China

**C. Li**

China

**Song-Lin Li**

China

**Min Li**

Hong Kong

**Peng Li**

Macao

**Zhitao Liang**

Hong Kong

**Jyh-Fei Liao**

Taiwan

**Min Yee Lim**

Singapore

**Zhixiu Lin**

Hong Kong

**Houwen Lin**

China

**Guido Lingua**

Italy

**Jianping Liu**

China

**Cun-Zhi Liu**

China

**Yuanyan Liu**

China

**Fang Liu**

China

**Xusheng Liu**

China

**Guanghua Lu**

China

**Aiping Lu**

Hong Kong

**Hongmei Luo**

China

**Yangchao Luo**

USA

**Dik Lung Ma**

Hong Kong

**Yiming Ma**

USA

**Sameh Magdeldin**

Japan

**Kouji Miyazaki**

Japan

**Andrea Mombelli**

Switzerland

**Jin Mou**

Canada

**Nagalakshmi Nadiminty**

USA

**Stephen Ng**

Hong Kong

**Tzi Bun Ng**

Hong Kong

**Grace Njoroge**

Kenya

**Taketo Okada**

Japan

**Bulbul Pandit**

USA

**Yong-Ki Park**

South Korea

**Kishore Polireddy**

USA

**Eamonn Quigley**

Ireland

**Saravanan Rajendrasozhan**

Saudi Arabia

**Sathishkumar Ramalingam**

India

**Giancarlo Ripabelli**

Italy

**Jianhui Rong**

Hong Kong

**Kuber Sampath**

USA

**Johannes Saukel**

Austria

**Michael Seidman**

USA

**Masoomeh Shams-Ghahfarokhi**

Iran

**Hongcai shang**

China

**Pang-Chui Shaw**

Hong Kong

**Jie Shen**

USA

**C. Shen**

USA

**Johannah Shergis**

Australia

**Ming-Jyh Sheu**

Taiwan

**Rong Shu**

China

**Mario Simirgiotis**

Chile

**Jingyuan Song**

China

**Hutcha Sriplung**

Thailand

**Rowan Stringer**

Switzerland

**Hermann Stuppner**

Austria

**Shi-Bing Su**

China

**Zhibo Sun**

China

**Stephen Cho Wing Sze**

Hong Kong

**Erna Sziksz**

Hungary

**Hiroyuki Tada**

Japan

**Hor Yue Tan**

Malaysia

**Mihai Udrescu**

Romania

**Ruchan Uslu**

Turkey

**Fons van de Loo**

The Netherlands

**Leo Van Griensven**

The Netherlands

**Sivarama Prasad Vinjamury**

USA

**Jian-Bo Wan**

Macao

**Chao Wan**

Hong Kong

**Jianhua Wang**

China

**Ping Wang**

USA

**XinLuan Wang**

China

**Ying Wang**

Macao

**YuMing Wang**

China

**Shiwei Wang**

Hong Kong

**Jon Wardle**

Australia

**Elaine Wat**

Australia

**Natthida Weerapreeyakul**

Thailand

**Zehuai Wen**

China

**Qingping Wen**

China

**Martin Wiemers**

Germany

**Elizabeth Williamson**

UK

**Wendy Wong**

Hong Kong

**Chun-Kwok Wong**

Hong Kong

**Xiaohe Xiao**

China

**Pei-shan Xie**

China

**Liwei Xie**

USA

**Yan Xu**

Canada

**Huanbin Xu**

USA

**Ru Yan**

China

**Rong-Sen Yang**

Taiwan

**Xianwen Yang**

China

**Meihua Yang**

China

**Zhi Yao**

UK

**Huiyu Yao**

China

**Tao Yi**

Hong Kong

**Sheng Yin**

China

**Jia Yongliang**

China

**Zhixian Yu**

USA

**Patrick Ying-kit Yue**

Hong Kong

**Eunice Yuen**

UK

**Sze-chung Yuen**

Macao

**Su Zeng**

China

**Hong Lin Zhai**

China

**Qingwen Zhang**

Macao

**Ge Zhang**

Hong Kong

**Claire Shuiqing Zhang**

Australia

**Xu Zhang**

Canada

**Yanjun Zhang**

China

**Yan Zhao**

China

**Jing Zhao**

Macao

**Zhongzhen Zhao**

Hong Kong

**Zhen Zheng**

Australia

**Guo-qing Zheng**

China

**Yan Zhou**

China

**Hongmei Zhu**

Macao

**Guo-Yuan Zhu**

China

**Wioletta Zukiewicz-Sobczak**

Poland

**Jian-Ping Zuo**

China

